# Letter from Dr. Harris to the New-York Editor

**Published:** 1841

**Authors:** 


*Extract of a Letter from Dr. Harris to the New-York Editor.*

Baltimore, Dec. 3, 1840.

My Dear Sir,

Knowing the lively interest you take in every thing that tends to the advancement of our favourite science, I embrace the present opportunity to inform you that our *College of Dental Surgery* is now in successful operation. Our class is small, but perhaps as large as we had a right to expect, and is made up of young gentlemen that would do honor to any profession.

My colleague, Dr. Hayden, commenced upon osteology, which he recommended to the class as constituting a subject that should first engage their attention. He has endeavoured to inculcate new views upon this subject, and we believe, not without success. He repudiates all of John Hunter's ideas of bone "nibblers and modelers," with John Bell's bone modelers, the absorbents;—and Haller's notions of arterial action as the agent employed in shaping and forming the cavities in the bones,—and moreover, most of the other theories, which, Meckle says, "are either vague or false."

He asserts, that the formation of all of the osseous tissues, is regulated by a system of immutable laws, known only to Him who created and impressed upon them the indelible marks of his ineffable goodness, wisdom and power.

This position he sustains by referring the whole process of ossification to that period, the parturient state, in which, for nine months, all the phenomena of ossification are carried on, and to a certain extent perfected, without the aid of the exhalents, or absorbents, or the vital forces from which the absorbents, according to Bichat, derive their power.

These views were sustained by facts and illustrations that seemed to set doubt at rest. Yet, lest it might be contended that the vital forces derived from the mother through the medium of the umbilical cord, were sufficient to qualify the absorbents with all their attributes, he appealed to the process of ossification in the oviparous animals, where, during the entire period of incubation, no influence, either direct or indirect, could, by a possibility, have been derived from the parent of the eggs. Yet the whole process of ossification, with the attendant phenomena, were, as shown by strikingi llustrations, precisely the same, and, apparently regulated by the same laws as that of ossification in all viviparous animals.

The illustrations were the most complete and satisfactory in the bones of the tortoise, or *testudo terrestris major*, which he exhibited.

This is but an imperfect sketch of the Doctor's argument in support of his views on this subject. It was not only able and ingenious, but seemed very conclusive. But with the abilities of Dr. H. you are already acquainted, so that of them it is unnecessary for me to speak. As a theorist and practitioner in our profession, you are aware that he ranks among the very first.

Professor Bond sustains the chair of special pathology and therapeutics in a very able manner. As a teacher, I very much question whether he has any superior. While his manner is easy, and free from every thing like embarrassment, he possesses in an eminent degree, the happy faculty of so impressing what he says upon the minds of his hearers, as not easily to be forgotten.

In his first lecture, he began by defining Life, considered physically, to be the assemblage of effects produced by a given organization:—*Health* to consist in order and regularity in the developement of these effects:—Disease, disorder, or irregularity, in the accomplishment of one or more of them. Physiology had regard to the succession of acts which collectively constituted life. Hygien, to the means by which health may be preserved; and Pathology, to diseases and their cure.

The causes of disease he explained as external and internal. The external, all those morbific causes which are not dependent for their existence upon our organization—internal, such as are engrafted upon our constitution by inheritance, or are produced spontaneously within us by the action of one part of our being upon another, or by the natural decay of organs. Further divisions into local and general—predisposing and exciting—specific, &c.

He then proceeded to consider the agency of the atmosphere in producing disease.

Professor Baxley's first lecture was on the elementary principles of animal organization, and was highly interesting, but of this I have not time to give you even an outline. As a teacher of anatomy, Dr. B. has acquired a justly deserved high reputation, and we were fortunate in obtaining the services of so distinguished a man.

The school having gone into successful operation, there is now no question as to the future. The scheme, at first, was thought by many to be chimerical, and it was believed that the institution would never go into operation. This prevented many residing in remote parts of the country from attending the first course. Indeed, I have received letters from a number since it has commenced, stating their desire to attend, but who were unwilling to incur the expense of coming here until they should ascertain that the school was actually in operation. There will no longer be grounds for such apprehension. The scheme was not projected without counting the cost; the school was chartered by an enlightened legislature, disposed to elevate a useful profession to the rank it should occupy, and rescue it from the hands of charlatans. It is *in being* with a degree of success equalling our most sanguine expectations, and is destined, we do not doubt, to take position among the useful institutions of the country.

